# Plasmon resonances management of meta-mirror for combining radar cross section (RCS) reduction and specular reflection with frequency selectivity

**DOI:** 10.1038/s41598-021-97403-3

**Published:** 2021-09-09

**Authors:** Ali Pesarakloo, Alireza Oruji

**Affiliations:** Electromagnetic Waves Propagation Laboratory, School of Electrical Engineering, Iran University of Science and Technology, 1684613114 Tehran, Iran

**Keywords:** Electrical and electronic engineering, Electronics, photonics and device physics

## Abstract

In this paper using Plasmon Resonances Management (PRM), a bi-functional meta-mirror is proposed in which, the meta-mirror can obtain two opposite properties in two different frequency ranges. In this method, an anisotropic unit cell with polarization conversion property is modified to have two plasmon resonances in both symmetric and anti-symmetric planes in a specific frequency. This allows the unit cell to have the property of unchanged polarization in that frequency. The meta-mirror is composed of this modified unit cell and its mirror as a chessboard arrangement and the incident wave on the meta-mirror is reflected as in-phase in that specific frequency i.e. specular reflection, while as out-of-phase in other frequencies i.e. RCS reduction. The designed meta-mirror in this paper demonstrates the RCS reduction in two side-bands from 4 to 9 GHz and 10.8 to 14.8 GHz while behaving as a specular reflection in the frequency around 10 GHz.

## Introduction

Reflective metasurfaces referred to as Meta-Mirrors are periodic or quasi-periodic structures composed of resonant subwavelength elements (meta-atom) mounted on the dielectric substrate with subwavelength thickness and backed with the metallic surface^1^. When the Electromagnetic (EM) waves irradiate the meta-mirror, by engineering the geometric parameters of the subwavelength elements such as the shape, size and orientation the desired phase discontinuity can be achieved such that the scattered waves have anomalous properties with respect to the metal plate^2^. The exotic properties that can be achieved by meta-mirrors are such as high-sensitivity sensing^3–9^, polarization conversion^10–12^, anomalous reflection^13,14^, RCS reduction^15,16^, RCS enhancement^17–19^, power divider^20^. One of the methods for dispersion tailoring of scattered wave is the use of electric and magnetic plasmon resonances^21,22^. Recently, several works have focused on utilizing this method in meta-mirrors to realize specific functions^3–11,15,16^. In^3–9^ Jing Chen et al. proposed different periodic array structures to achieve a very strong magnetic plasmon resonance in metamaterials and metasurfaces for high-sensitivity sensing and light absorption enhancement applications. In^10^ Hongya Chen et al. proposed an ultra-wideband Polarization Conversion Meta-mirror (PCM) that is designed using a unit cell structure composed of oblique V-shaped and cut-wire resonators. These resonators generate four plasmon resonances in four different frequencies and cause the complete rotation of linear polarization to its orthogonal one in these four frequencies. As a result, this structure can produce an ultra-wideband linear polarization rotator. In^15,16^ the PCM is used for mono-static RCS reduction. The term "reduction" indicates the difference between the RCS of the structure and a metal plate of the same dimensions. The unit of RCS is dBsm (dB square meter) and for RCS reduction is dB. There, the unit cell structure that is used for PCM is mirrored that produce two unit-cell with “0” and “1” elements owing to their 0 and π reflection phase responses. Then the macro-cells of these two unit cells can be arranged like a chessboard or randomly using the optimization algorithms to generate a structure with RCS reduction property.

In some applications, such as the RCS reduction of the radar system antennas, the planar structure located as the ground plane of the antenna must function as mirror reflection in its operating frequency. In addition, it must operate as the RCS reduction in two wide bands' frequencies outside the operating frequency to make the antenna invisible. Another application is to reduce the RCS of military equipment with the ability to detect by friendly radars. Here also a planar structure must be mounted on the equipment to achieve mirror reflection in the operating frequency of the friendly radar, as well as to reduce RCS in the two sidebands. To the best of our knowledge, this type of meta-mirror just demonstrated in^23^. The two meta-atoms with optimized metallic patterns are used as the binary coding elements to realize an out-of-phase function in two side-bands, while the in-phase function is in the center frequency window. Experimentally it is demonstrated that RCS reduction can be achieved by the proposed meta-mirror with randomly distributed meta-atoms in two side-bands from 7.5 to 9.5 GHz and 11.6 to 15 GHz, while in the selected center frequency window around 10.7 GHz, a high-efficient specular reflection property is realized^23^.

In this paper the PRM is used to achieve the combining of RCS reduction and specular reflection with frequency selectivity i.e. a bi-functional meta-mirror. In this method, a modified unit cell structure of the PCM is designed such that the arrangement of plasmon resonances in frequency bandwidth produce a null in the specified frequency window in the cross-polarization rotation plot of the unit cell. When this unit cell and its mirror are arranged like a chessboard, the resultant structure can achieve considerable RCS reduction in the two outside bands, while behaving as an ideal mirror and performing specular reflection in the selected frequency window. Both simulation and experimental measurement results indicate that the proposed meta-mirror can achieve RCS reduction in two side-bands from 4 to 9 GHz and 10.8 to 14.8 GHz while behaving as a specular reflection in the frequency around 10 GHz. It should be noted that the bandwidth obtained from the proposed method is broader than the bandwidth in^23^.

## Design procedure

### Basic unit cell design

A well-known unit cell that is used in Ultra Wide-Band (UWB) PCM is composed of a double-head arrow array with a backing metal ground sheet that is separated by a dielectric substrate. Different types of this unit-cell are proposed in the literature^10,11^.

**Figure 1:**
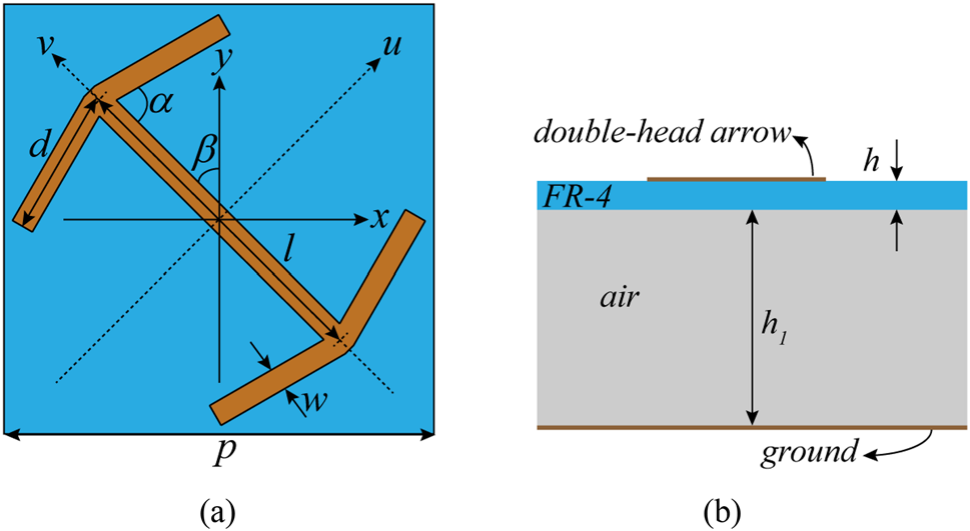
Schematic of basic unit-cell (**a**) top view (**b**) side view.

The basic unit cell that is used in this literature also is a double-head arrow, as shown in Fig. 1. This unit-cell is mounted on an FR-4 dielectric with thickness 0.25 mm,$\varepsilon_{r} = 4.3$ and $\tan \delta = 0.025$, and separated from the ground sheet by the air dielectric with thickness 7.5 mm. The reason for choosing this type of compound substrate is to be able to take advantage of both the low cost and availability of the FR-4 substrate and the low effective dielectric constant, because the lower the effective dielectric constant of the structure, the higher the bandwidth of the structure performance. The periodicity of the meta-mirror unit cell is 15 mm. The other parameters of the double-head arrow structure are: $l = 12\;{\text{mm}},\;d = 5\;{\text{mm}},\;w = 0.8\;{\text{mm}},\;\alpha = 75^{ \circ } ,\; \, \beta = 45^{ \circ }$. The thickness of metallic patterns are $t = 0.018\;{\text{mm}}$.

To see how PRM should be done to have polarization conversion in the entire desired frequency range, the plasmon resonances of the considered double-head arrow structure are studied first. To do this, we use the resonances study of the Split-Ring-Resonators (SRRs) at normal incidence, which was done in^24–28^. There, it was concluded that if the electric field oscillates parallel to the gap of the SRR, it can couple to several plasmon resonances in different frequencies. The first two resonances of the SRR structure are stronger, that the first is of magnetic resonance in which a current loop is induced on the SRR which can be modeled as LC circuit^28^. The gap of the SRR is regarded as a capacitance, and the ‘U’ as a single winding of an inductance loop^25^. This resonance also called LC resonance. The second is of electric resonance that is associated with a plasmon excitation in the ‘U’ shaped wire^26^. Additionally, in^26,27^ it was shown that excitation of the LC-resonance does not necessarily require a gap. The resonance remains present in the limit of a vanishing gap where the SRR consists of a single wire piece.

Two unit vectors are defined to describe the orientation of the unit cell structure: u along the symmetry axis of the structure and v perpendicular to u, as in Fig. 1. The double-head arrow structure supports “symmetric” and “anti-symmetric” modes, which are excited by electric-field along u and v axes, respectively. In the symmetric mode, two plasmon resonances can occur. The current distribution in the annular path consists of both arms of the V-shaped part and the gap between them approximate a magnetic resonator of $(\omega_{LC} )_{sym} = \frac{1}{{\sqrt {L_{1} C_{1} } }}$. The current distribution in both arms of the V-shaped part approximates an electric resonator of length $2d \approx \frac{{(\lambda_{eff} )_{sym} }}{2}$ where $\lambda_{eff}$ is the effective wavelength. In the anti-symmetrical mode also these two types of plasmon resonances occur. The current distribution in the annular path consists of one arm of V-shaped in the lower part, cut-wire, one arm of V-shaped in the upper part and the gap between them approximate a magnetic resonator of $(\omega_{LC} )_{asym} = \frac{1}{{\sqrt {L_{2} C_{2} } }}$. The current distribution in one arm of V-shaped in the lower part, cut-wire and one arm of V-shaped in the upper part approximate an electric resonator of length $d + l + d$, and therefore the electric resonance occurs at $d + l + d \approx \frac{{(\lambda_{eff} )_{asym} }}{2}$.

**Figure 2:**
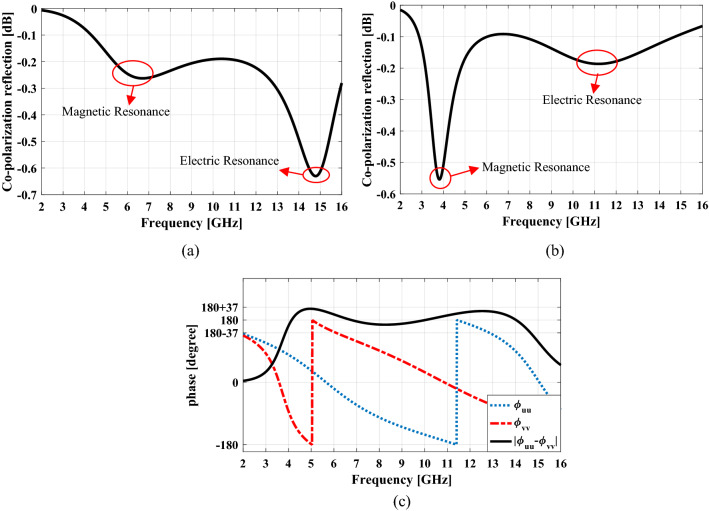
The four eigenmodes of the double-head arrow under normal incidence: (**a**) v-polarized case and (**b**) u-polarized case (**c**) the phase of the reflected wave in u-polarization, v-polarization and the phase difference between them.

**Figure 3:**
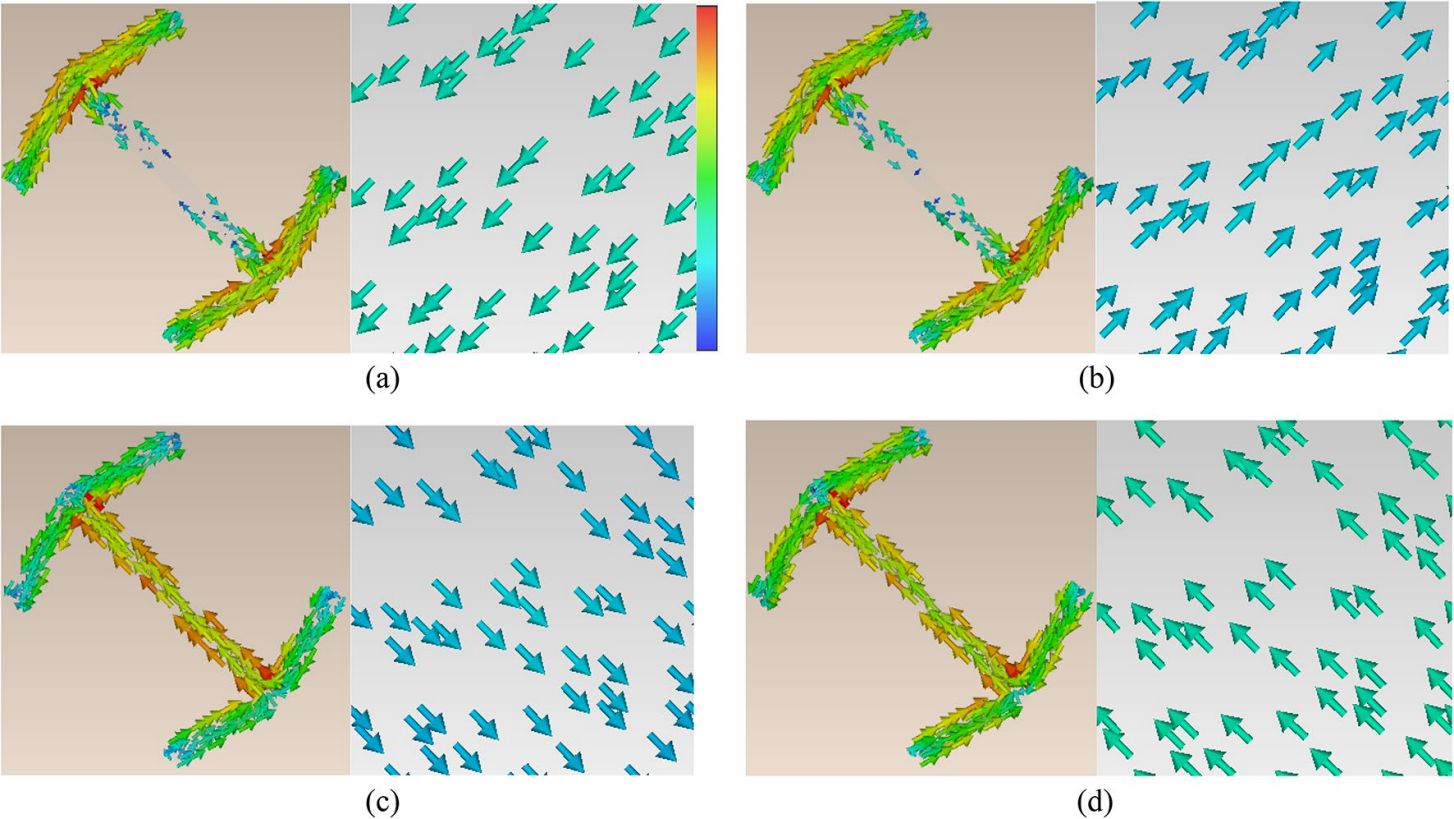
Surface current distributions on the resonator part and ground sheet of the basic unit cell. (**a**) 6.7 GHz eigenmode (i), (**b**) 14.8 GHz eigenmode (iii), (**c**) 3.8 GHz eigenmode (ii), and (**d**) 11.2 GHz eigenmode (iv).

Therefore it can be concluded that the double-head arrow structure has electric and magnetic plasmon resonances when it is excited by electric field along u or v axis. In order to understand the number and related frequency of plasmon resonances of the double-head arrow structure for an incident wave with certain polarization, the reflection amplitude and phase of the unit cell are studied by CST Microwave Studio simulation^29^. The co-polarization reflection versus frequency for the excitation of the electric field along u and v axes are shown in Fig. 2a,b, respectively. It can be seen that two eigenmodes are excited in both u and v-polarized case. The two eigenmodes with lower frequency, are of magnetic resonance and the two other ones are of electric resonance. This can be concluded from the resonance length of electric and magnetic resonators. To clarify the type of resonance in each eigenmode, the distribution of surface current on the resonator part and ground sheet for two type plasmon resonances in symmetric and anti-symmetric modes has been monitored in Fig. 3.

As can be seen, the electric and magnetic resonances are generated by parallel and anti-parallel surface currents induced along the resonator part and the metallic ground sheet, respectively^21^.

When a plane wave with *y-* (*x-*) polarization impinges on the unit cell structure in a wide frequency bandwidth, four plasmon resonances can be excited since both *u-* and *v-*components exist simultaneously. In all of these resonance frequencies the *y-* (*x-*) polarized incident wave is rotated 90° by the unit cell and converts completely to *x-*(*y-*) polarized one because at each of these frequencies, according to Fig. 2c the electric field components along *u-*axis and *v-*axis are reflected with 180° phase difference relative to each other. On the other hand, because the Co-polarization reflection relation is as follows:

$$r_{xx} (or\; \, r_{yy} ) = 10\log \left[ {\frac{{A_{uu} e^{{j\phi_{uu} }} + A_{vv} e^{{j\phi_{vv} }} }}{2}} \right]^{2} .$$

As long as the phase difference $\left| {\phi_{uu} - \phi_{vv} } \right|$ is within $180^{ \circ } \pm 37^{ \circ }$, the Co-polarization reflection is below − 10 dB or the Cross-polarization reflection above − 0.5 dB. Therefore, the polarization conversion can be achieved in a wide frequency bandwidth that includes these resonant frequencies. When this unit cell is mirrored to *x-* or *y-*axis, the mirrored structure has 90° polarization rotation but the inverse of the former one i.e. the *y-* (*x-*) polarized incident wave converts to -*x-* (-*y-*) polarized one.

**Figure 4:**
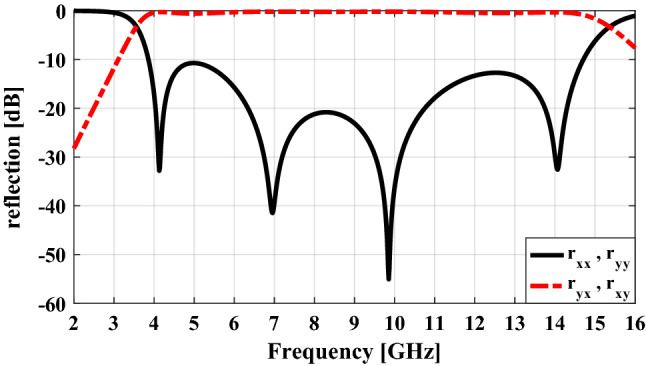
Simulated results of the co- and cross-polarization reflections.

To verify the above-mentioned content, the co- and cross-polarization reflections of the unit cell are plotted in Fig. 4 for both *y-* and *x-*polarized normal incident wave using the CST Microwave Studio^29^ with periodic boundary conditions in *x-* and *y-*directions and open conditions along the *z-*direction. As can be seen there are four nulls in the plot of co-polarization reflection versus frequency (i.e. the polarization conversion efficiency nearly 100%) that are resulted from the four plasmon resonances of the unit cell and the frequencies at which these nulls have occurred are almost the same as the resonant frequencies of the unit cell. As a result, ultra wide-band polarization conversion from 4 to 16 GHz (with the criteria − 10 dB in co-polarization reflection) can be achieved under both *x-* and *y-*polarized waves at normal incidence.

**Figure 5:**
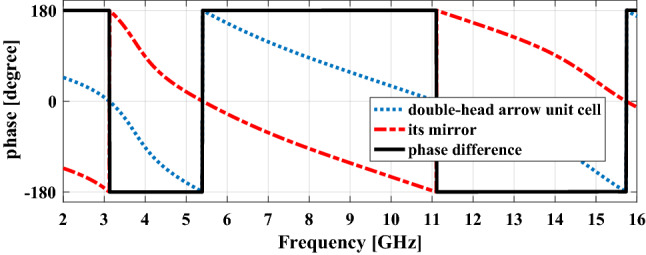
The phase of cross-polarization reflection for unit cell in Fig. 2 and its mirror and the phase difference between them.

As mentioned earlier, the infinite periodic of the basic unit cell and its mirror have cross-polarization reflection with the same magnitude but 180° phase difference, as shown in Fig. 5. Therefore, if the macro-cells of these two unit cells are arranged in a chessboard pattern next to each other the reflected waves from these two sub-sections will counteract each other in a bore-sight direction and finally, RCS reduction will be achieved.

From the above, it can be concluded that in order to have polarization conversion or RCS reduction in a certain frequency range, PRM must be done in such a way that several plasmon resonances are distributed over the entire frequency range, each in different frequency.

### Proposed unit cell design

**Figure 6:**
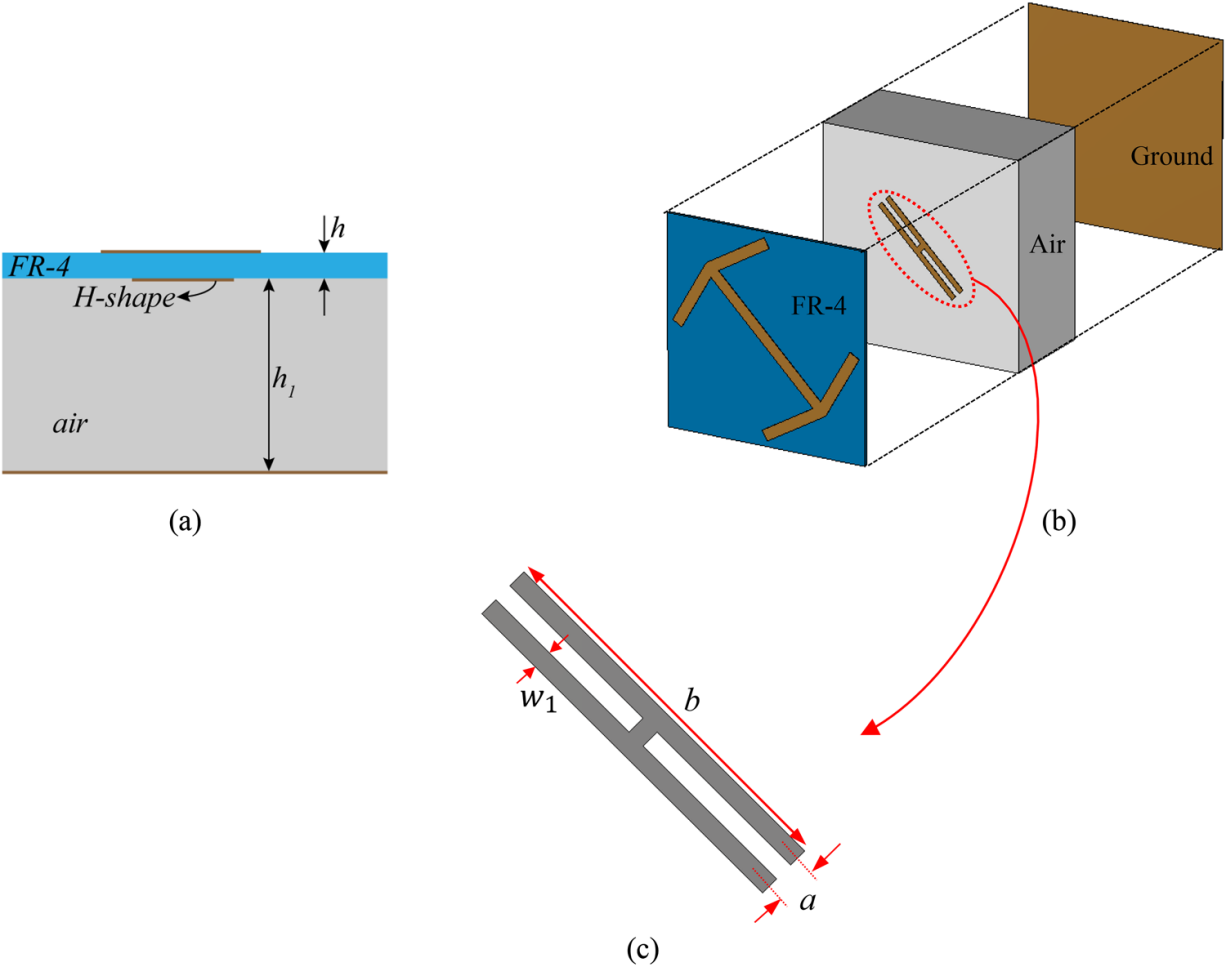
(**a**) Side view of the proposed unit cell, (**b**) the composition of proposed unit cell (**c**) H-shape.

**Figure 7:**
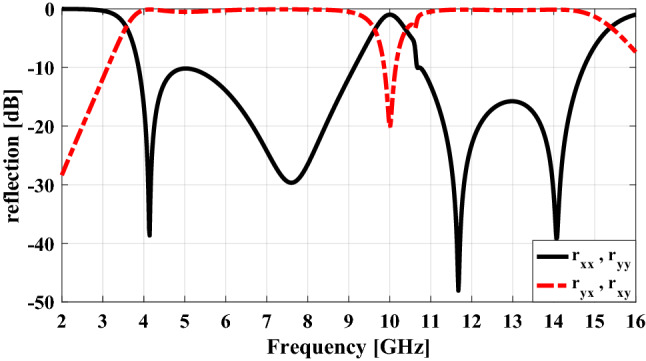
Simulated results of the co- and cross-polarization reflections for proposed unit cell.

Now to add the specular reflection in a certain frequency to the recent RCS reduction structure, the basic unit cell must be modified to have full co-polarization reflection (null in cross-polarization reflection) in that frequency and reasonable cross-polarization reflection in the rest of the bandwidth. In other words, the polarization rotation should not occur in that specific frequency. To achieve this, PRM must be done in such a way that in the frequency ranges with RCS reduction, several plasmon resonances are distributed throughout that range, but in the frequencies with specular reflection, there are two plasmon resonances at the same frequency to counteract each other. To realize this, an H shape structure is added to the basic unit cell as shown in Fig. 6. The dimension of the H shape structure are: $a = 0.8\;{\text{mm}},\;b = 4\;{\text{mm}},\;w_{1} = 0.4$. The co- and cross-polarization reflection diagrams for the proposed unit cell are shown in Fig. 7. As can be seen one null in the cross-polarization reflection is created in frequency $f = 10\;{\text{GHz}}$.

**Figure 8:**
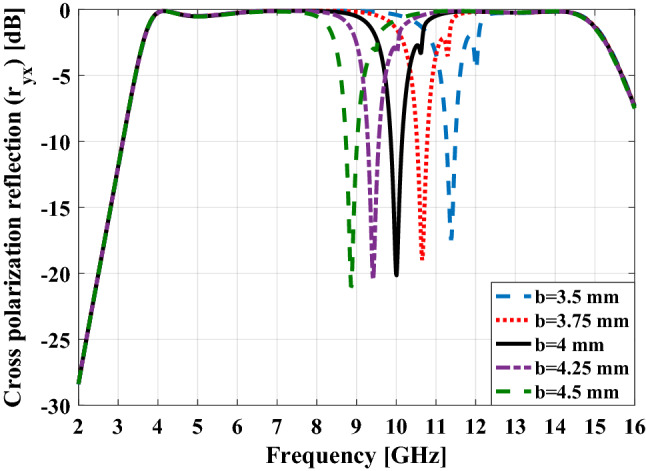
The cross-polarization reflection for proposed unit cell versus frequency in different b values.

The excellent property that the proposed unit cell offers is the possibility of moving the frequency location of the null in the cross-polarization reflection diagram by changing the dimensions of the H shaped structure. This property is shown in Fig. 8.

**Figure 9:**
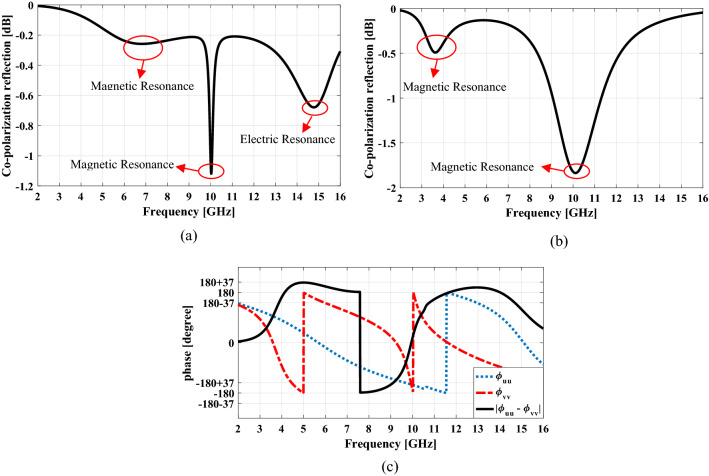
The eigenmodes of the proposed unit cell under normal incidence: (**a**) v-polarized case and (**b**) u-polarized case, (**c**) the phase of reflected wave in u-polarization, v-polarization and the phase difference between them.

**Figure 10:**
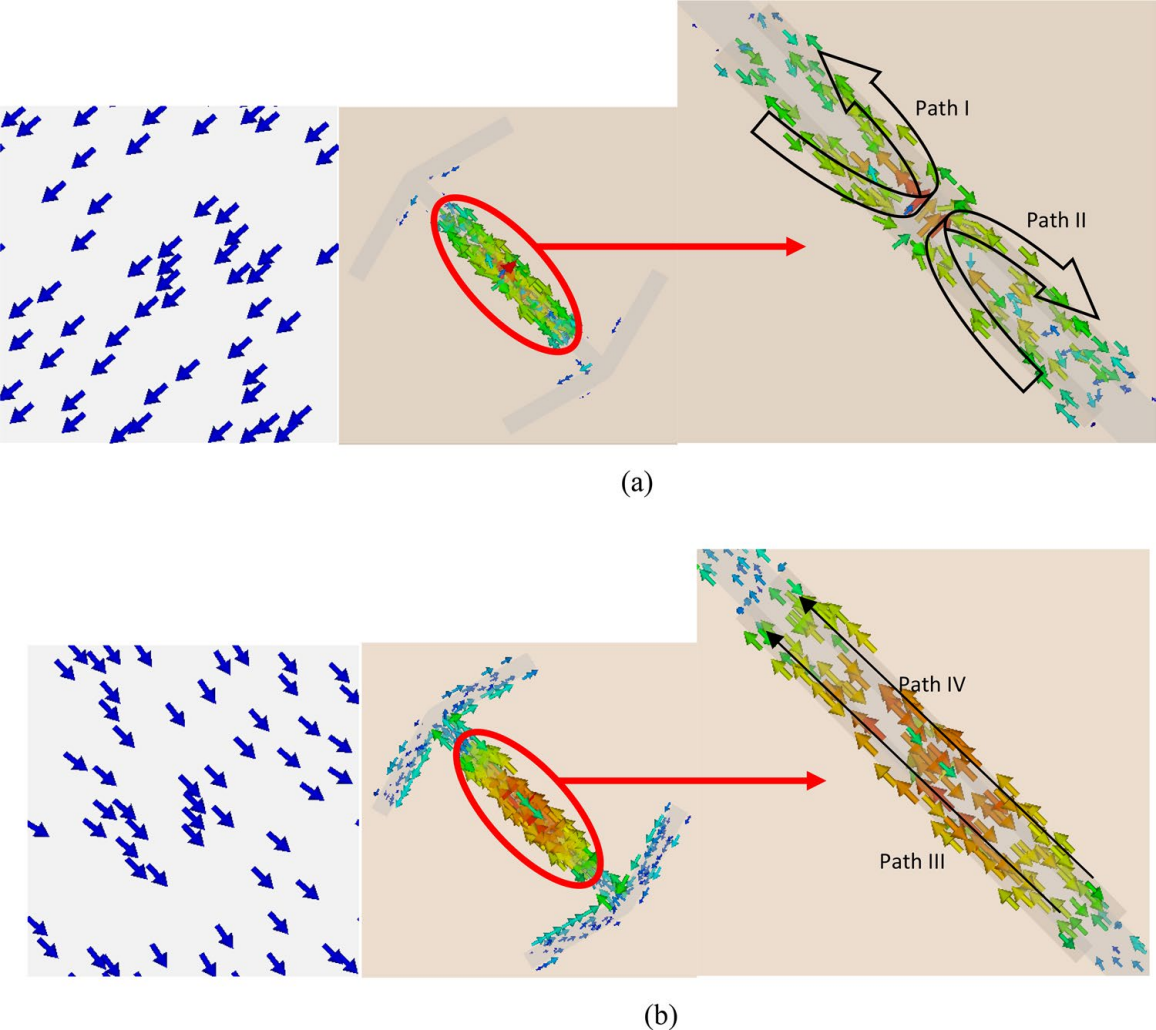
The surface current distribution on the ground sheet and resonator part of the proposed unit cell at frequency 10 GHz for (**a**) u-polarization and (**b**) v-polarization.

To better understand how the null happens, like the basic unit cell the plasmon resonances of the proposed structure are examined. The co-polarization reflection versus frequency for the excitation of the electric field along *u* and *v* axes for the proposed unit cell are shown in Fig. 9a,b, respectively. It can be seen that there are two plasmon resonances in the frequency of about 10 GHz for *u-* and *v-*polarization. The reason is that in the *u-*polarization the SRR-like structure of the H-shape (as shown in Fig. 10a) causes LC resonance and in the *v-*polarization the single wire structures of the H-shape (as shown in Fig. 10b) also causes LC resonance^26,27^ so that these two resonances occur at the same frequency. According to Fig. 9c, these two resonances cause the electric field components along u-axis and v-axis are reflected in-phase that causes a null in the cross-polarization reflection of the proposed unit cell when it is illuminated by a plane wave with *y-* (*x-*) polarization. The reason for the wide resonance around the frequency of 10 GHz in *u-*polarization is the proximity of the magnetic resonance frequency of the H-shaped structure and the electrical resonance of the basic unit-cell and as a result their overlap. The surface current distribution on the proposed unit cell at frequency 10 GHz for both *u-* and *v-*polarization is shown in Fig. 10. As can be seen, for incident wave with *u-*polarization the electric current flow in paths I and II as shown in Fig. 10a, while for incident wave with *v-*polarization it flow in paths III and IV as shown in Fig. 10b.

## Meta-mirror design and simulation

**Figure 11:**
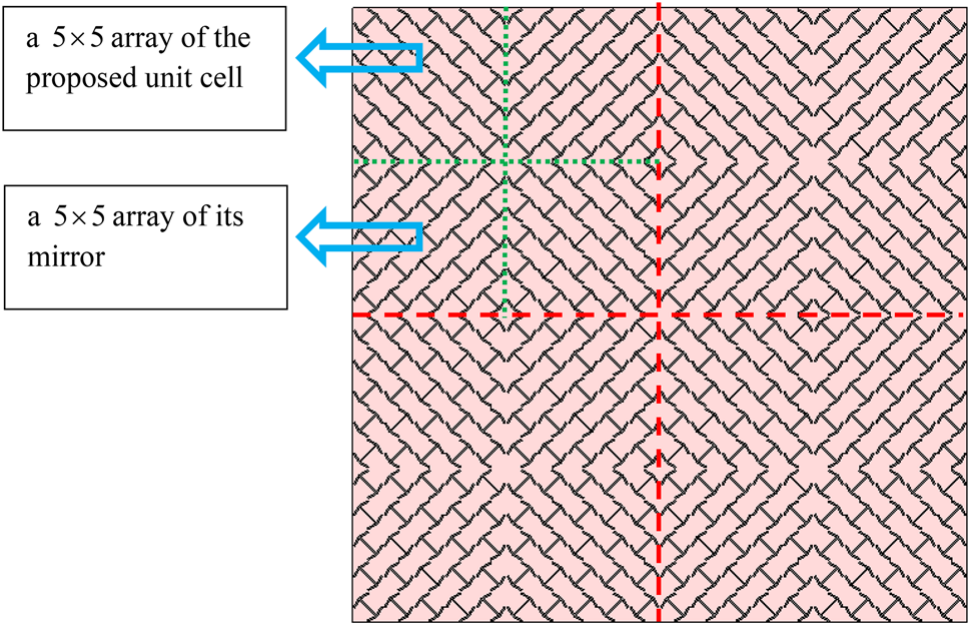
The full structure formed of the proposed unit array and its mirror. (The H-shape is on the back of the substrate and is not visible.)

Now to verify the RCS reduction and specular reflection properties of the proposed meta-mirror, full-wave simulations are performed in CST Microwave Studio^29^. The simulated structure is such that a $5 \times 5$ array of the proposed unit cell and a $5 \times 5$ array of its mirror are placed side by side in a checkerboard pattern which form a macro-cell. Finally, a $2 \times 2$ array of this macro-cell is arranged as Fig. 11.

**Figure 12:**
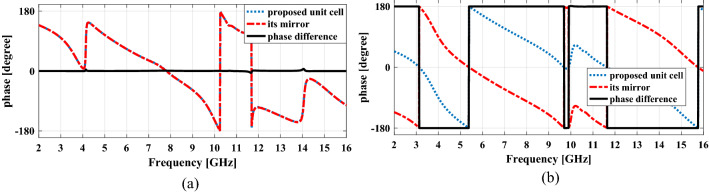
The phase of (**a**) co-polarization reflection for proposed unit cell and its mirror and the phase difference between them. (**b**) Cross-polarization reflection for proposed unit cell and its mirror and the phase difference between them.

Now, the mechanism of the meta-mirror is explained. When a plane wave with *x-* or *y-*polarization is normally incident on the meta-mirror structure, in the frequency ranges on both sides of the frequency 10 GHz, the reflection of the unit cell and its mirror is of the cross-polarization reflection type, so according to Fig. 12b, there is a 180° phase difference between them, which cancel each other and thus RCS reduction occur. But in the frequency 10 GHz, the reflection of the unit cell and its mirror is of the co-polarization reflection type, so according to Fig. 12a, they are in-phase which add each other and thus specular reflection occur.

**Figure 13:**
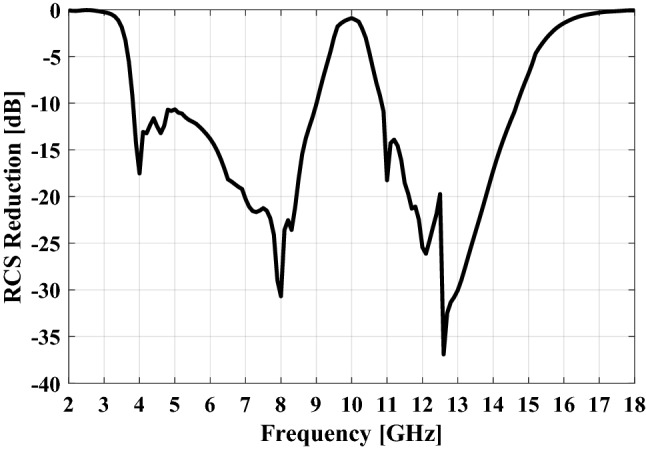
The RCS reduction pattern of proposed meta-mirror for normal incidence.

**Figure 14:**
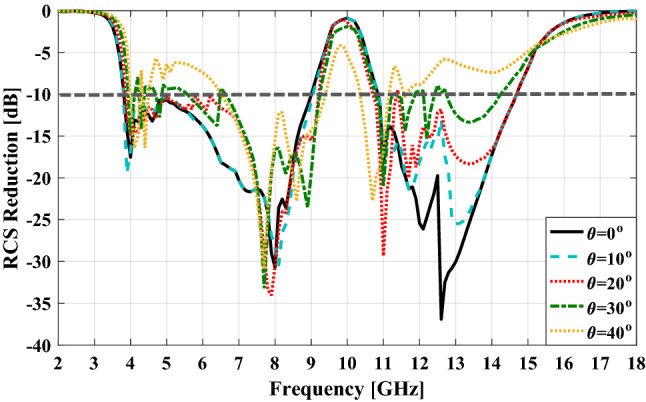
The RCS reduction of the proposed meta-mirror for different incident angles.

Now, the extraction of the RCS reduction diagram in the CST STUDIO software^29^ is explained. First a plane wave with *x-* or *y-*polarization is normally incident on the meta-mirror structure and a PEC ground with the same size. Then the RCS of these two structures versus frequency are obtained and finally the normalized RCS of meta-mirror with respect to the PEC ground is evaluated that is shown in Fig. 13. As can be seen, from 4 to 9 GHz and 10.8 to 14.8 GHz the meta-mirror operated as an RCS reduction structure while behaving as a specular reflection in the frequency around 10 GHz. The simulation results of the proposed meta-mirror in different oblique incident angles up to 40° are demonstrated in Fig. 14. As can be seen the meta-mirror has a relatively stable angular performance for incident angles up to 30°, with a nearly unchanged RCS reduction bandwidth and a high reflection window.

**Figure 15:**
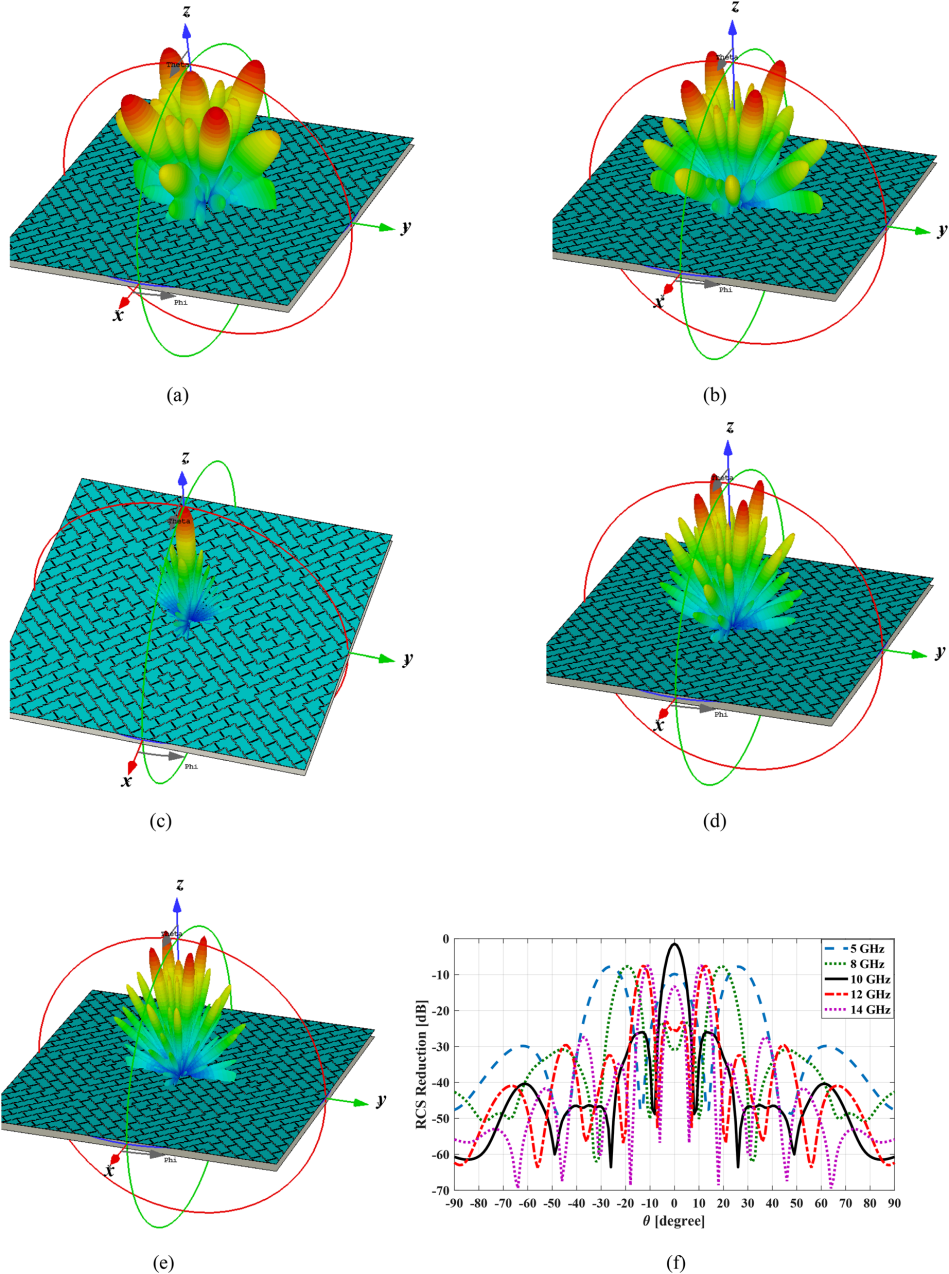
The 3D scattering pattern in (**a**) f = 5 GHz, (**b**) f = 8 GHz, (**c**) f = 10 GHz, (**d**) f = 12 GHz and (**e**) f = 14 GHz, (**f**) the 2D scattering pattern in $\varphi = 45^{ \circ }$ plane to show the amount of RCS reduction in different frequencies.

Figure 15a–e show the simulated three-dimensional (3D) scattering patterns of the meta-mirror for the normal *y-*polarized incident angle at 5 GHz, 8 GHz, 10 GHz, 12 GHz and 14 GHz, respectively. The patterns are normalized to the RCS of a metal plate with the same dimension as the proposed structure. Figure 15f show the simulated 2D scattering patterns of the meta-mirror in $\varphi = 45^{ \circ }$ plane for different frequencies to indicate the amount of RCS reduction.

## Fabrication and experiment

**Figure 16:**
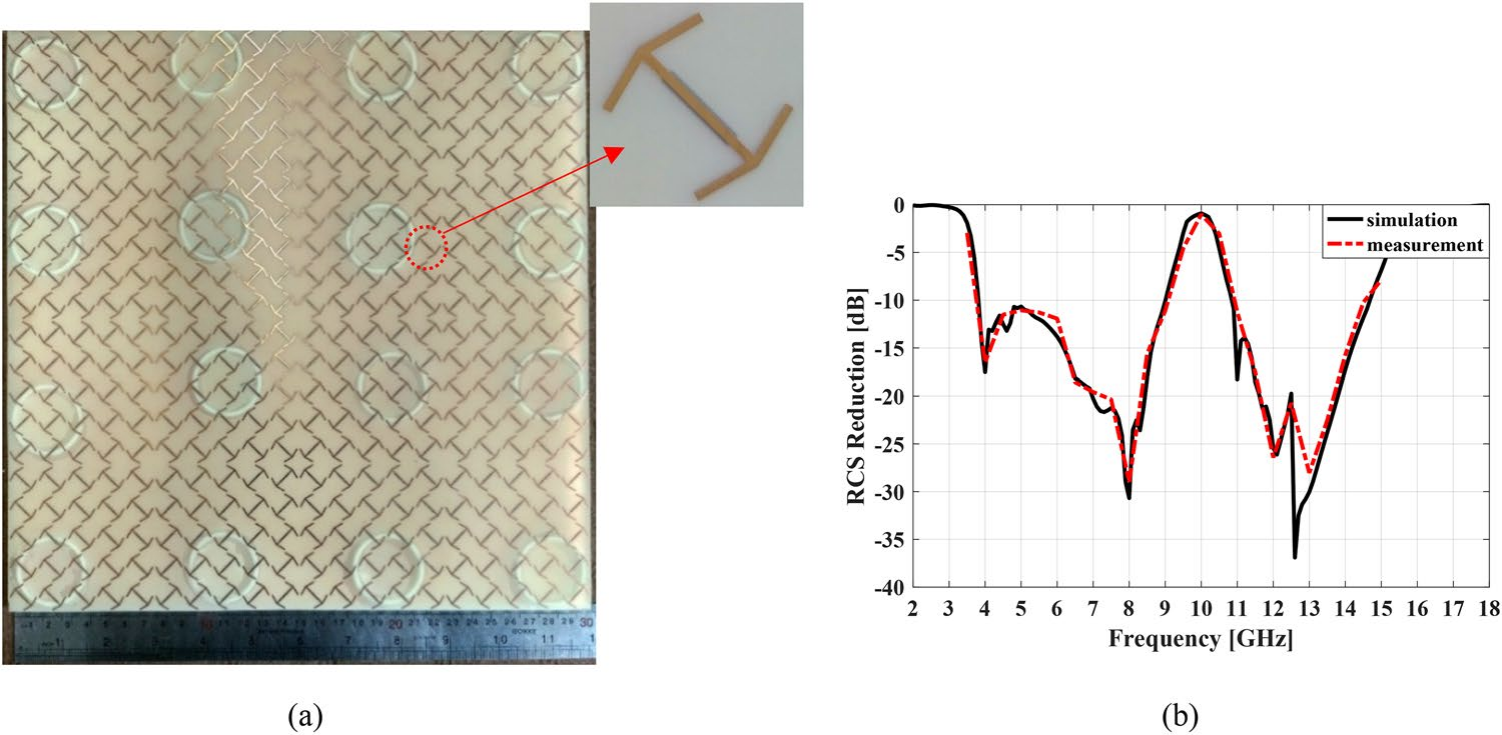
(**a**) The prototype of proposed meta-mirror, (**b**) simulation and measurement results of the proposed meta-mirror versus frequency for normal incidence.

Figure 16a depicts the fabricated meta-mirror structure that its overall dimensions is $320 \times 320\;{\text{mm}}^{2}$. The fabricated structure composed of an ultra-thin low cost commercially available FR-4 substrate placed on top of a ground plane. The double-head arrow unit cells print on the top layer of the FR-4 substrate with $h = 0.25\;{\text{mm}}$ thickness while the H-shapes print on the bottom layer of the substrate. This layer is spaced by an air gap of $h_{1} = 7.5\;{\text{mm}}$ from the ground copper sheet using spacers.

In Fig. 16b the measured RCS reduction versus frequency for manufactured structure at the normal incident wave as well as the simulation results are shown. As can be seen, there is a reasonable agreement between them.

## Conclusions

In this paper, a meta-mirror combining specular reflection in a specific frequency and wideband RCS reduction in two sidebands was proposed based on PRM. In this method, an anisotropic polarization conversion unit cell is designed to generate two plasmon resonances in symmetric and anti-symmetric planes in a specific frequency. The meta-mirror is a checkered combination of this unit cell and its mirror one that when interacting with incident EM waves it can achieve no polarization conversion and therefore specular reflection in a specific frequency. Both the full-wave simulation and experimental test on a fabricated prototype validate that the proposed meta-mirror can achieve the RCS reduction in two side-bands from 4 to 9 GHz and 10.8 to 14.8 GHz while behaving as a specular reflection in the frequency around 10 GHz.

## Data Availability

The datasets generated during and/or analysed during the current study are available from the corresponding author on reasonable request.
